# Association of Smoking With Patient Characteristics and Outcomes in Small Cell Lung Carcinoma, 2011-2018

**DOI:** 10.1001/jamanetworkopen.2022.4830

**Published:** 2022-03-30

**Authors:** Jeng-Sen Tseng, Chun-Ju Chiang, Kun-Chieh Chen, Zhe-Rong Zheng, Tsung-Ying Yang, Wen-Chung Lee, Kuo-Hsuan Hsu, Yen-Hsiang Huang, Tsang-Wu Liu, Jiun-Yi Hsia, Gee-Chen Chang

**Affiliations:** 1 Division of Chest Medicine, Department of Internal Medicine, Taichung Veterans General Hospital, Taichung, Taiwan; 2 College of Medicine, National Chung Hsing University, Taichung, Taiwan; 3 Institute of Biomedical Sciences, National Chung Hsing University, Taichung, Taiwan; 4 College of Medicine, National Yang Ming Chiao Tung University, Taipei, Taiwan; 5 Institute of Epidemiology and Preventive Medicine, College of Public Health, National Taiwan University, Taipei, Taiwan; 6 Taiwan Cancer Registry, Taipei, Taiwan; 7 Division of Pulmonary Medicine, Department of Internal Medicine, Chung Shan Medical University Hospital, Taichung, Taiwan; 8 School of Medicine, Chung Shan Medical University, Taichung, Taiwan; 9 Institute of Medicine, Chung Shan Medical University, Taichung, Taiwan; 10 Department of Life Sciences, National Chung Hsing University, Taichung, Taiwan; 11 Division of Critical Care and Respiratory Therapy, Department of Internal Medicine, Taichung Veterans General Hospital, Taichung, Taiwan; 12 National Institute of Cancer Research, National Health Research Institutes, Miaoli, Taiwan; 13 Division of Thoracic Surgery, Department of Surgery, Chung Shan Medical University Hospital, Taichung, Taiwan

## Abstract

**Importance:**

Small cell lung carcinoma (SCLC) is uncommon in individuals who have never smoked (never-smokers). The related epidemiologic factors and prognosis remain unclear.

**Objective:**

To assess the epidemiologic factors, clinical characteristics, and outcomes of SCLC in never-smokers.

**Design, Setting, and Participants:**

A retrospective cohort study was conducted using data from the national Taiwan Cancer Registry, which was inaugurated in 1979 and maintains standardized records of patients’ characteristics and clinical information for all individuals with cancer. Patients with cytologically or pathologically proven lung cancer were included for analysis. The study obtained data on patients from January 1, 1996, to December 31, 2018; data analysis was conducted from January 1, 1996, to December 31, 2019.

**Exposures:**

Clinical characteristics and outcomes of smokers and never-smokers with SCLC.

**Main Outcomes and Measures:**

Clinical characteristics for comparison included age at diagnosis, sex, performance status, tumor stage, and treatment. The main outcome parameter was overall survival of patients with SCLC from 2011 to 2018.

**Results:**

From 1996 to 2018, a total of 225 788 patients had diagnosed lung cancer; 141 654 patients (62.7%) were men; mean (SD) age was 67.55 (12.58) years. The numbers of both patients with newly diagnosed lung cancer and those with SCLC increased until 2009 by 111.5% for lung cancer and 118.5% for SCLC. Thereafter, lung cancer cases grew in number, but SCLC cases did not; hence, the percentage of patients with SCLC decreased from 9.3% in 2009 to 6.3% in 2018. From 2011 to 2018, the percentage of never-smokers increased significantly among all patients with lung cancers (from 49.9% in 2011 to 60.2% in 2018) and among those with lung adenocarcinomas (from 64.1% in 2011 to 70.9% in 2018) (both *P* < .001). However, the percentage of never-smokers appeared to vary little in the SCLC population: 15.5% in 2011 and 16.1% in 2018 (*P* = .28). The median overall survival was significantly longer in patients with adenocarcinoma vs SCLC (adjusted hazard ratio, 0.32; 95% CI, 0.31-0.33; *P* < .001). Compared with smokers with SCLC, never-smokers with SCLC tended to include more older patients (age ≥70 years: 492 [57.3%] vs 2242 [44.8%]), more women (274 [31.9%] vs 322 [6.4%]), more individuals with a poor performance status (Eastern Cooperative Oncology Group performance status ≥2: 284 [33.1%] vs 1261 [25.2%]) and stage IV cancer (660 [76.9%] vs 3590 [71.8%]), and more patients without treatment (203 [23.7%] vs 626 [12.5%]). Furthermore, never-smokers, particularly men, experienced a shorter survival rate (adjusted hazard ratio, 1.10; 95% CI, 1.00-1.20; *P* = .04) compared with the other groups.

**Conclusions and Relevance:**

The findings of this cohort study suggest that the decrease in the percentage of patients with SCLC was associated with increased lung cancers of other histologic types, with no substantial decrease in the number of those with SCLC.

## Key Points

**Question:**

Do patient characteristics of smokers and never-smokers differ among patients with small cell lung carcinoma (SCLC)?

**Findings:**

In this cohort study examining 225 788 patients with lung cancer, among patients with SCLC, there were more older individuals, more women, more patients with a poor performance status and in an advanced stage of cancer, and more patients who did not receive treatment among never-smokers than among smokers. Never-smokers, particularly men, experienced worse outcomes.

**Meaning:**

The findings of this study suggest that clinical characteristics and outcomes of patients with SCLC differ between smokers and never-smokers.

## Teaser

This cohort study examines characteristics of patients with small cell lung carcinoma who had a history of cigarette smoking vs never smoking between 1996 and 2018.

## Introduction

Lung cancer is the leading cause of cancer-related death worldwide.^1,2^ Because lung cancer is a heterogeneous disease, patients with different histologic types may have various predisposing risk factors, clinical presentations, and treatment options.^3,4^ In addition, the histologic classification could serve as an important prognostic factor.^5^ Adenocarcinoma is currently the most common histologic type of lung cancer, and studies across countries have reported decreases in the incidence of small cell lung carcinoma (SCLC).^6,7^ A decrease in the prevalence of cigarette smoking is a likely cause of the decreases.^7^

Cigarette smoking is a well-known risk factor for several types of cancer and results in an increasing number of deaths.^8^ Cigarette smoking is not only associated with lung cancer^9^ but also affects the evolution, pathologic features, molecular characteristics, efficacy of treatment, and overall outcomes.^10,11,12,13^ However, more studies have reported an increase in lung cancer in never-smokers, especially in women, individuals who are Asian, and those with adenocarcinoma.^14^ Several risk factors other than cigarette smoking have been reported; of these, radon exposure has been shown to be one of the leading causes of lung cancer in never-smokers.^15^ Although the global prevalence of cigarette smoking is decreasing, the number of lung cancer cases continues to increase.^1,8^ Therefore, a study of lung cancer in never-smokers in terms of its tumorigenesis, characteristics, and outcomes is warranted.

The outcome of patients with lung cancer has substantially improved owing to advances in understanding its molecular pathogenesis and in new treatments for the disease, but the benefits are mostly confined to patients with lung adenocarcinoma.^4^ Treatment for SCLC continues to be mainly chemotherapy. The most important advancement is adding immunotherapy to chemotherapy, which lengthens the survival time.^16,17^

Small cell lung carcinoma mostly occurs in smokers, but some studies have reported SCLC cases in never-smokers.^18,19,20,21,22,23,24,25^ Moreover, the clinical characteristics and mutation profiles of SCLC may differ between smokers and never-smokers.^21,23^ Because most of these studies are retrospective and have a limited number of patients, their results are not consistent. Whether smoking status is associated with patient outcome remains unclear. Herein, we report on a study we conducted with a nationwide population in Taiwan that aimed to explore the epidemiologic factors, clinical characteristics, and outcomes of SCLC in never-smokers.

## Methods

### Patients and Study Cohorts

This retrospective cohort study used data from the national Taiwan Cancer Registry. The database was inaugurated in 1979 and keeps standardized records of patients’ characteristics and clinical information on all cancer cases in Taiwan.^12,26,27,28^ Detailed information on the cigarette smoking status of patients with lung cancer has been in the database since 2011. To be eligible for our study, patients with lung cancer needed to have cytologic or pathologic evidence showing clear classification of histologic types. We analyzed the data on lung cancer occurring from January 1, 1996, to December 31, 2018, for the epidemiologic study of all lung cancers and SCLC. The main outcome parameter was overall survival of patients with SCLC from 2011 to 2018. Furthermore, to study the changes in patient characteristics and the outcomes of all patients with lung cancer, adenocarcinoma, and SCLC, we analyzed data from January 1, 2011, to December 31, 2018. We also evaluated the association of smoking status with the outcomes and clinical characteristics of patients with SCLC with known smoking, staging, and survival status. Data analysis was conducted from January 1, 1996, to December 31, 2019. The patient selection and analysis flowchart is shown in eFigure 1 in the Supplement. This study was approved by the Research Ethics Committee of the National Taiwan University (NTU-REC No.202101HM030), with waiver of informed consent owing to the lack of personal information and use of secondary data in the study. The Strengthening the Reporting of Observational Studies in Epidemiology (STROBE) reporting guideline for observational studies was used in the revision of this article.

### Data Records

Clinical data used for analysis included age at diagnosis, sex, histologic types, performance status, tumor stage, smoking status, Eastern Cooperative Oncology Group performance status, and vital status. Never-smokers were defined as those who had never smoked in their lifetime; otherwise, patients were classified as smokers. Data on lung cancer in the Taiwan Cancer Registry before 2018 were recorded according to the 7th edition of the American Joint Committee on Cancer staging system before 2018 and, after that, according to the 8th edition of the American Joint Committee on Cancer staging system.^29,30^ We categorized patients as having stages I to III or stage IV cancer, which was not affected by the updated staging system.

### Statistical Analysis

The overall survival time was defined as the time from the date of diagnosis to the date of death or until the last date of follow-up. Survival status was evaluated using the national death certificate database from the Department of Statistics, Ministry of Health and Welfare, Taiwan, and it was updated until December 31, 2019. Records were excluded if the date of death was unknown. The χ^2^ test was used to evaluate the association between categorical variables. Overall survival was estimated using Kaplan-Meier analysis and compared using the log-rank test. The association between clinicopathologic variables and outcomes was assessed using Cox proportional hazards regression models. Hazard ratios (HRs) and 95% CIs were calculated in both the univariate and multivariate models. Two-sided statistical significance was set at *P* < .05. All analyses were performed using SAS, version 9.4 statistical software (SAS Institute Inc).

## Results

### Overall Lung Cancer and SCLC Cases

We included a total of 225 788 patients with lung cancer newly diagnosed from 1996 to 2018 in our analysis (141 654 [62.7%] men; 84 134 [37.3%] women); mean (SD) age was 67.55 (12.58) years. The epidemiologic trend over time of all lung cancers and SCLC cases is shown in eFigure 2 in the Supplement. The number of new lung cancer cases increased by 111.5% from 1996 to 2009. The number of newly diagnosed cases of SCLC was 454 in 1996 and 992 in 2009, corresponding to an increment of 118.5%. The trends toward an increase in cases of SCLC and of all lung cancers were comparable. Small cell lung carcinoma accounted for approximately 9% of all lung cancers.

The number of lung cancers increased by 39.6% from 2009 to 2018. However, the number of SCLC cases did not change substantially during the same decade. Rather, the percentage of SCLC cases declined from 9.3% in 2009 to 6.3% in 2018.

### Change in Smoking Status

**Figure 1.**
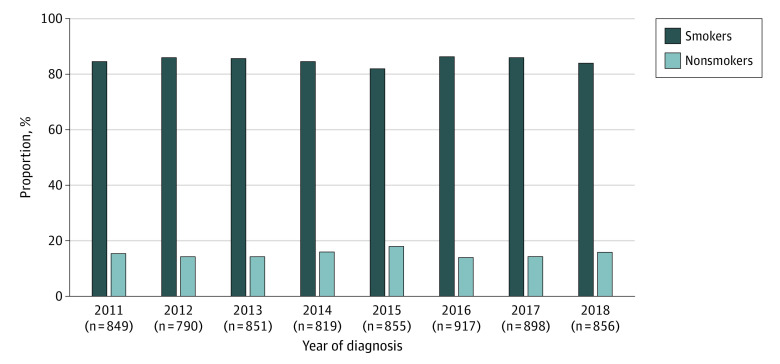
Changes in Smoking Status Among Patients With Small Cell Lung Carcinoma From 2011 to 2018

We analyzed a total of 96 565 patients with lung cancer with known smoking status from 2011 to 2018, including 6835 individuals with SCLC. From 2011 to 2018, the percentage of never-smokers increased significantly among all patients with lung cancers (from 49.9% in 2011 to 60.2% in 2018) and among those with lung adenocarcinomas (from 64.1% in 2011 to 70.9% in 2018) (both *P* < .001) (eFigure 3 in the Supplement). In contrast, no such change was found in the SCLC population, with never-smokers accounting for 15.5% of SCLC cases in 2011 and 16.1% in 2018 (*P* = .28) (Figure 1).

### Patient Characteristics and Outcomes

We analyzed patients with lung cancer diagnosed from 2011 to 2018 to compare the differences in characteristics and outcomes of different histologic types. Patients without clear tumor staging data were excluded. A total of 83 590 patients were included and the results are summarized in Table 1.

**Table 1.** Patient Characteristics and Outcomes of All Lung Cancer, Adenocarcinoma, and SCLC Cases From 2011 to 2018^a^

Factor	No. (%)		
	All lung cancer (N = 83 590)	Adenocarcinoma (n = 56 511)	SCLC (n = 6338)
Age, y			
<70	47 505 (56.8)	35 229 (62.3)	3267 (51.5)
≥70	36 085 (43.2)	21 282 (37.7)	3071 (48.5)
Sex			
Female	34 896 (41.7)	30 039 (53.2)	655 (10.3)
Male	48 694 (58.3)	26 472 (46.8)	5683 (89.7)
Smoking status			
Never-smokers	42 158 (50.4)	35 668 (63.1)	858 (13.5)
Smokers	35 598 (42.6)	17 904 (31.7)	5000 (78.9)
Unknown	5834 (7.0)	2939 (5.2)	480 (7.6)
ECOG PS			
0-1	45 133 (54.0)	32 956 (58.3)	3011 (47.5)
≥2	13 943 (16.7)	8054 (14.3)	1562 (24.6)
Unknown	24 514 (29.3)	15 501 (27.4)	1765 (27.8)
Stage			
I-III	33 424 (40.0)	22 275 (39.4)	1707 (26.9)
IV	50 166 (60.0)	34 236 (60.6)	4631 (73.1)
Treatment			
No	12 126 (14.5)	5055 (9.0)	1261 (19.9)
Yes (any)	71 464 (85.5)	51 456 (91.1)	5077 (80.1)
Outcome			
5-y OS rate, %	26.0	33.0	4.8
Median survival time (95% CI), mo^b^	18.2 (18.0-18.5)	27.9 (27.4-28.3)	7.2 (7.0-7.5)

Abbreviations: ECOG PS, Eastern Cooperative Oncology Group performance status; HR, hazard ratio; OS, overall survival; SCLC, small cell lung carcinoma.

^a^ Comparison was conducted between lung adenocarcinoma and SCLC (patient characteristics by χ^2^ test and survival time by log-rank test and Cox proportional hazards regression model). All differences were significant at *P* < .001.

^b^ Adenocarcinoma vs SCLC: adjusted HR, 0.32 (95% CI, 0.31-0.33).

Regarding clinical characteristics, more patients were aged 70 years or older in the SCLC group (3071 [48.5%]) vs the adenocarcinoma group (21 282 [37.7%]) (*P* < .001). The SCLC population compared with the adenocarcinoma group also contained more men (5683 [89.7%] vs 26 472 [46.8%]), more smokers (5000 [78.9%] vs 17 904 [31.7%]), more patients with a poor performance status (Eastern Cooperative Oncology Group performance status ≥2) (1562 [24.6%] vs 8054 [14.3%]), and more patients with stage IV disease (4631 [73.1%] vs 34 236 [60.6%]) (*P* < .001 for all subgroups). More patients with SCLC did not receive antineoplastic treatments (1261 [19.9%] vs 5055 [8.9%]; *P* < .001), and those with adenocarcinoma lived significantly longer than patients with SCLC (27.9 months; 95% CI, 27.4-28.3 months vs 7.2 months; 95% CI, 7.0-7.5 months) (adjusted HR, 0.32; 95% CI, 0.31-0.33; *P* < .001) (Table 1; eFigure 4 in the Supplement).

### Association of Smoking Status With SCLC Outcomes

Data on patients with SCLC diagnosed from 2011 to 2018 with known tumor staging data, smoking status, and clear survival follow-up results were analyzed. The overall survival of the entire SCLC population was 7.8 months (95% CI, 7.6-8.0 months); for those with stages I to III disease, survival was 14.1 months (95% CI, 13.3-15.0 months), and those with stage IV disease survived 6.3 months (95% CI, 6.0-6.6 months) (eFigure 5 in the Supplement). The median age at diagnosis of SCLC in smokers was 68 years (range, 29-106) and in never-smokers was 72 years (range, 26-99). As reported in Table 2, there were more never-smokers aged 70 years or older compared with smokers (492 [57.3%] vs 2242 [44.8%]). Never-smokers with SCLC were more likely to be women (274 [31.9%] vs 322 [6.4%]), have a poor performance status at diagnosis (Eastern Cooperative Oncology Group performance status ≥2) (284 [33.1%] vs 1261 [25.2%]), and have stage IV disease (660 [76.9%] vs 3590 [71.8%]) (*P* < .001 for all subgroups).

**Table 2.** Univariate Analysis of Characteristics Between Smokers and Never-Smokers With SCLC From 2011 to 2018^a^

Factor	No. (%)		
	All SCLC (n = 5858)	Smokers (n = 5000)	Never-smokers (n = 858)
Age, y			
<70	3124 (53.3)	2758 (55.2)	366 (42.7)
≥70	2734 (46.7)	2242 (44.8)	492 (57.3)
Sex			
Female	596 (10.2)	322 (6.4)	274 (31.9)
Male	5262 (89.8)	4678 (93.6)	584 (68.1)
ECOG PS			
0-1	2995 (51.1)	2641 (52.8)	354 (41.3)
≥2	1545 (26.4)	1261 (25.2)	284 (33.1)
Unknown	1318 (22.5)	1098 (22.0)	220 (25.6)
Stage			
I-III	1608 (27.4)	1410 (28.2)	198 (23.1)
IV	4250 (72.6)	3590 (71.8)	660 (76.9)
Treatment			
No	829 (14.2)	626 (12.5)	203 (23.7)
Yes			
Any	5029 (85.8)	4374 (87.5)	655 (76.3)
Chemotherapy	4625 (79.0)	4052 (81.0)	573 (66.8)
Radiotherapy	2023 (34.5)	1839 (36.8)	184 (21.4)
Operation	183 (3.1)	150 (3.0)	33 (3.8)

Abbreviations: ECOG PS, Eastern Cooperative Oncology Group performance status; SCLC, small cell lung carcinoma.

^a^ Comparison between smokers and never-smokers by χ^2^ test. All differences were significant at *P* < .001.

**Figure 2.**
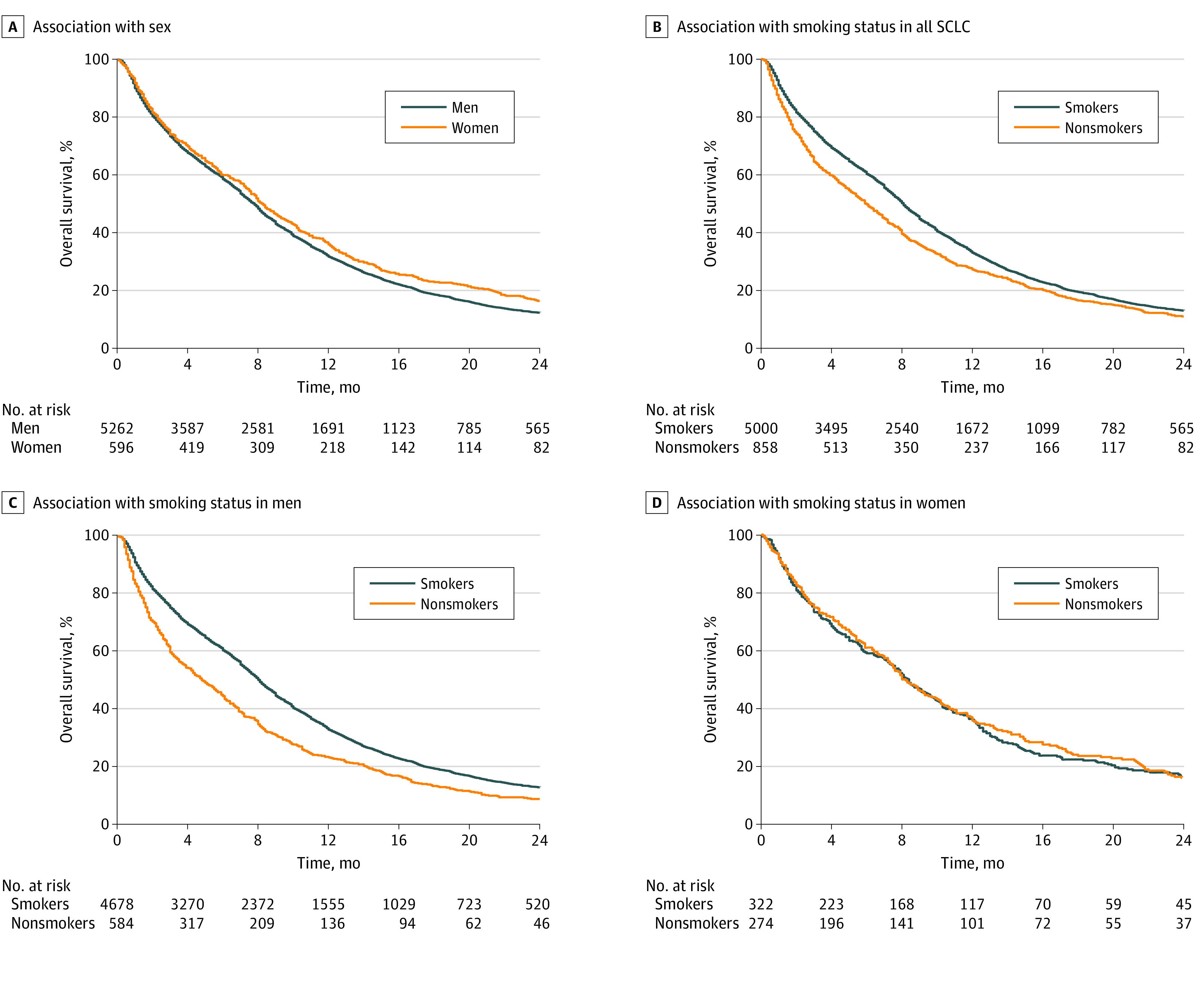
Association of Sex and Smoking Status With Overall Survival of Patients With Small Cell Lung Carcinoma (SCLC) From 2011 to 2018 Association of sex with all SCLC (A), association of smoking status with all SCLC (B), association of smoking status with SCLC in men (C), and association of smoking status with SCLC in women (D).

More never-smoker patients with SCLC did not receive antineoplastic treatments (203 [23.7%] vs 626 [12.5%]; *P* < .001), and lower proportions of never-smokers had a history of chemotherapy (573 [66.8%] vs 4052 [81.0%]) and radiotherapy (184 [21.4%] vs 1839 [36.8%]) compared with smokers. In view of the significant association that was found between sex and smoking behavior, we further evaluated the association of sex and smoking status with patient outcome, with results are presented in Figure 2. Among the entire SCLC population, overall survival time was 8.3 months (95% CI, 7.6-9.1 months) for women and 7.8 months (95% CI, 7.5-8.0 months) for men (*P* = .01). The overall survival time was 6.0 months (95% CI, 5.3-6.7 months) for never-smokers and 8.1 months (95% CI, 7.8-8.3 months) for smokers (*P* < .001). In the stratified analyses regarding sex and smoking status, we found that the association of smoking behavior with patient outcome was mainly limited to men. In the male SCLC population, never-smokers had a significantly shorter survival time than smokers (4.8 months; 95% CI, 4.0-5.7 months vs 8.1; 95% CI, 7.8-8.3 months; *P* < .001). In contrast, outcomes of women were comparable between smokers (8.4; 95% CI, 7.3-9.6 months) and never-smokers (8.1 months; 95% CI, 7.6-9.5 months) (*P* = .79). There was no significant difference in overall survival time between male and female smokers. Similar trends in the differences of survival time between smokers and never-smokers were observed in both stages I to III and stage IV disease (eFigure 6 in the Supplement).

The baseline characteristics of men and women with SCLC are reported in eTable 1 and eTable 2 in the Supplement. Among men, never-smokers were older, more had a poor performance status and stage IV disease, and more had not received antineoplastic treatment compared with smokers. In contrast, baseline characteristics of women with SCLC were similar between smokers and never-smokers. Multivariate analyses were performed to adjust for age, performance status, tumor stage, and history of treatments. Among men, never-smokers experienced a shorter survival time than smokers (adjusted HR, 1.10; 95% CI, 1.00-1.20; *P* = .04). No significant association between smoking status and patient outcomes was found in women (adjusted HR, 0.87; 95% CI, 0.73-1.03; *P* = .10). The comparisons of characteristics between female and male never-smokers are presented in eTable 3 in the Supplement. Male never-smokers were older, and more had not received treatment than female never-smokers.

## Discussion

It remains unclear whether the characteristics and outcomes of SCLC differ between smokers and never-smokers. Interactions between genes and the environment are presumably important in the cause of cancer.^31^ However, not all lung cancers can be explained by etiologic factors. One well-known example is how smoking status affects the clinical presentations and genetic profiles of lung cancer. Such effects are diverse among patients with different races and ethnicities and histologic types.^32,33^ Because SCLC rarely occurs in never-smokers, our knowledge of these patients comes mainly from retrospective studies with limited case numbers.^18,19^ In Taiwan, we conducted our study using a nationwide cohort, which could provide a meaningful description of the epidemiologic factors, clinical characteristics, and outcomes of SCLC in never-smokers. Compared with a previous study in Taiwan analyzing the SCLC population from 2004 to 2006,^34^ there was not much improvement in survival time. This finding is consistent with the limited advancements noted in SCLC treatment before immunotherapy was available.^16,17^

Analyses from both the Surveillance, Epidemiologic, and End Results database (1973-2002) and the Thames Cancer Registry database (1970-2007) have suggested a decreasing incidence of SCLC.^6,7^ Consistent with that projection, the percentage of SCLC cases among all lung cancers has also decreased in Taiwan, especially during the most recent decade. However, because the actual number of SCLC cases has not decreased on a yearly basis, the decrease in the percentage of SCLC was the result of an increase in lung cancer cases other than SCLC. Instead, the number of SCLC cases increased more than 60% from late 1990 to 2010. Although SCLC accounts for a small portion of all lung cancers, better understanding of the pathogenesis of SCLC can help improve its treatments.

Although lung cancers among never-smokers are more common among Asian patients,^14^ studies in the US and the UK both reported lung cancer cases in never-smokers,^35,36^ and most cohorts comprised individuals of White race. The study performed in the UK suggested that the incidence of lung cancers in never-smokers has remained stable or declined,^35^ but the outcomes associated with histologic types were not specified. In the present study, the percentage of never-smokers in the SCLC population remained steady, a finding that implied that the predisposing factors associated with tumorigenesis in never-smokers differ between lung adenocarcinoma and SCLC.^26^ Studies on non-Asian populations suggested that never-smokers accounted for only 2% to 6% of all SCLC cases.^23,25,37^ However, our present study, as well as studies from China and Korea, reported that 15% to 20% of patients with SCLC were never-smokers.^18,19^ Owing to the limitation of retrospective studies, data regarding radon exposure were not reported. In addition to lung adenocarcinoma,^32,38,39^ further studies on whether racial and ethnic differences also have effects on development of SCLC are required.

A recent study by Thomas et al^23^ evaluated the clinical and genomic characteristics of SCLC in never-smokers. Similar to our results, SCLC in never-smokers was found to include more older individuals, more women, and more patients with extensive-stage cancer. More than half of the patients in the Thomas et al^23^ study were of White race. Nine of 100 never-smoker patients with SCLC had genomic data, showing that never-smokers were characterized by a lower tumor mutation burden, fewer *TP53* mutations, and absence of mutational signatures associated with tobacco exposure. A study by Cardona et al^21^ included 10 smokers and 10 never/ever-smokers with SCLC. The main genetic mutations of the never/ever-smokers were *TP53* (80%), *RB1* (40%), *CYLD* (30%), and *EGFR* (30%), which differed from those of smokers. Another study on 19 patients with de novo SCLC, including 13 who were White, was reported by Varghese et al.^25^ Multiplex genotyping showed that the most common genetic alteration was *RB* loss, with 2 patients harboring *EGFR* mutations. In a Korean study by Sun et al,^19^ next-generation sequencing was performed with 28 never-smoker patients with SCLC. The investigators detected several genetic alterations, including *EGFR*, *TP53*, *RB1*, *PTEN*, *MET*, and *SMAD4*.

Based on the results of the studies discussed above and the present study, we suggest that SCLC in never-smokers possesses distinct clinical characteristics and may have different molecular mechanisms than SCLC in smokers. Owing to the limited case numbers, different detection methods, and heterogeneous populations of these studies, the exact genomic characteristics of these patients remain to be clarified. Radon exposure has been considered a risk factor for SCLC. Torres-Durán et al^20^ and others^40,41,42^ reported higher levels of residential radon exposure among never-smoker patients with SCLC. However, the causal relationship between radon and SCLC remains to be determined. Studies are required to identify other possible predisposing factors.

Multiple studies reported a poor prognosis for patients with SCLC. However, it remains uncertain whether there is a difference in outcome between smokers and never-smokers.^18,19,23^ Among patients with SCLC, men and never-smokers experienced a shorter overall survival time. The discrepancies in baseline performance status, tumor stage, and treatments account in part for the disparities. Our multivariate analysis and stratified analysis suggested that male never-smokers experienced the shortest survival time. In a subgroup analysis of the CASPIAN study, the overall survival benefit of add-on durvalumab to platinum plus etoposide was statistically significant in smokers (HR, 0.72; 95% CI, 0.58-0.91) but not in never-smokers (HR, 0.90; 95% CI, 0.40-2.11).^17^ In addition to the discrepancy in baseline characteristics, further studies are required to evaluate whether smoking status will influence the outcome.

### Limitations

This study has limitations. The major limitation is its retrospective design. The data were obtained from a registry database; hence, we cannot evaluate the efficacy of a particular treatment. Herein, we analyzed overall survival as the primary end point, which is unambiguous and can be used as a standard clinical outcome. Our patients had an equal chance of access to treatments because most chemotherapy regimens and radiotherapy for SCLC were reimbursed by the Taiwan National Health Insurance Administration during the study period. Another limitation of our study is the lack of radon exposure data for never-smokers with SCLC.

## Conclusions

The findings of this nationwide population-based cohort study in Taiwan suggest that the decrease in the percentage of SCLC cases was the result of increased lung cancers of other histologic types, with no decline in the number of SCLC cases. Approximately 15% of patients with SCLC were never-smokers, and the fraction of never-smokers remained steady. There were more older patients, more women, more individuals with a poor performance status and an advanced stage of cancer, and more patients without treatment among never-smokers with SCLC. Furthermore, never-smokers, particularly men, experienced worse outcomes.

## Notes

Supplement. **eTable 1.** Univariate Analysis of Characteristics Between Male Smokers and Never-Smokers With Small Cell Lung Carcinoma From 2011 to 2018 **eTable 2.** Univariate Analysis of Characteristics Between Female Smokers and Never-Smokers With Small Cell Lung Carcinoma From 2011 to 2018 **eTable 3.** Univariate Analysis of Characteristics Between Male and Female Never-Smokers With Small Cell Lung Carcinoma From 2011 to 2018 **eFigure 1.** Patient Selection and Analysis Flowchart **eFigure 2.** Epidemiological Trends of Overall Lung Cancer and Small Cell Lung Carcinoma (SCLC) From 1996 to 2018 (n = 225 788) **eFigure 3.** Changes in Smoking Status Among All Lung Cancer Patients (A) and Patients With Lung Adenocarcinoma (B) From 2011 to 2018 **eFigure 4.** Overall Survival of Patients With Small Cell Lung Carcinoma (SCLC) and Adenocarcinoma With Known Tumor Staging Data From 2011 to 2018 **eFigure 5.** Overall Survival of Patients With Small Cell Lung Carcinoma (SCLC) With Known Smoking Status and Tumor Staging Data From 2011-2018 **eFigure 6.** Overall Survival of Smokers and Never-Smokers With Small Cell Lung Carcinoma (SCLC) in Stage I-III (A) and IV (B)
