# Error in Affiliation

**DOI:** 10.1001/jamanetworkopen.2022.21124

**Published:** 2022-06-17

In the Original Investigation titled “Association of Smoking With Patient Characteristics and Outcomes in Small Cell Lung Carcinoma, 2011-2018,”^1^ published March 30, 2022, there was an error in an author affiliation. The affiliation for Jiun-Yi Hsia, MD, PhD, given as “Division of Pulmonary Medicine, Department of Internal Medicine, Chung Shan Medical University Hospital, Taichung, Taiwan” should have been “School of Medicine, Chung Shan Medical University, Taichung, Taiwan.” This article has been corrected.^1^
