# Supplementary material for: Association of Smoking With Patient Characteristics and Outcomes in Small Cell Lung Carcinoma, 2011-2018
Source: JAMA Netw Open. 2022 Mar 30;5(3):e224830. doi: 10.1001/jamanetworkopen.2022.4830 (PMC8968543; doi:10.1001/jamanetworkopen.2022.4830)
Supplement: Supplement. — eTable 1. Univariate Analysis of Characteristics Between Male Smokers and Never-Smokers With Small Cell Lung Carcinoma From 2011 to 2018 eTable 2. Univariate Analysis of Characteristics Between Female Smokers and Never-Smokers With Small Cell Lung Carcinoma From 2011 to 2018 eTable 3. Univariate Analysis of Characteristics Between Male and Female Never-Smokers With Small Cell Lung Carcinoma From 2011 to 2018 eFigure 1. Patient Selection and Analysis Flowchart eFigure 2. Epidemiological Trends of Overall Lung Cancer and Small Cell Lung Carcinoma (SCLC) From 1996 to 2018 (n = 225 788) eFigure 3. Changes in Smoking Status Among All Lung Cancer Patients (A) and Patients With Lung Adenocarcinoma (B) From 2011 to 2018 eFigure 4. Overall Survival of Patients With Small Cell Lung Carcinoma (SCLC) and Adenocarcinoma With Known Tumor Staging Data From 2011 to 2018 eFigure 5. Overall Survival of Patients With Small Cell Lung Carcinoma (SCLC) With Known Smoking Status and Tumor Staging Data From 2011-2018 eFigure 6. Overall Survival of Smokers and Never-Smokers With Small Cell Lung Carcinoma (SCLC) in Stage I-III (A) and IV (B) [file jamanetwopen-e224830-s001.pdf]

Supplementary Online Content

Tseng JS, Chiang CJ, Chen KC, et al. Association of smoking with patient characteristics and outcomes in small cell lung carcinoma, 2011-2018. *JAMA Netw Open*. 2022;5(3):e224830. doi:10.1001/jamanetworkopen.2022.4830

eTable 1. Univariate Analysis of Characteristics Between Male Smokers and Never-Smokers With Small Cell Lung Carcinoma From 2011 to 2018

eTable 2. Univariate Analysis of Characteristics Between Female Smokers and Never-Smokers With Small Cell Lung Carcinoma From 2011 to 2018

eTable 3. Univariate Analysis of Characteristics Between Male and Female Never-Smokers With Small Cell Lung Carcinoma From 2011 to 2018

eFigure 1. Patient Selection and Analysis Flowchart

eFigure 2. Epidemiological Trends of Overall Lung Cancer and Small Cell Lung Carcinoma (SCLC) From 1996 to 2018 (n = 225 788)

eFigure 3. Changes in Smoking Status Among All Lung Cancer Patients (A) and Patients With Lung Adenocarcinoma (B) From 2011 to 2018

eFigure 4. Overall Survival of Patients With Small Cell Lung Carcinoma (SCLC) and Adenocarcinoma With Known Tumor Staging Data From 2011 to 2018

eFigure 5. Overall Survival of Patients With Small Cell Lung Carcinoma (SCLC) With Known Smoking Status and Tumor Staging Data From 2011-2018

eFigure 6. Overall Survival of Smokers and Never-Smokers With Small Cell Lung Carcinoma (SCLC) in Stage I-III (A) and IV (B)

This supplementary material has been provided by the authors to give readers additional information about their work.

© 2022 Tseng JS et al. *JAMA Network Open.*

eTable 1. Univariate analysis of characteristics between male smokers and never-smokers with small cell lung carcinoma from 2011 to 2018

Factors	Male SCLC, No. (%) (n = 5262)	Smokers, No. (%) (n = 4678)	Never-smokers, No. (%) (n = 584)	*P* value[a]
Age				<.001
<70 years	2783 (52.9)	2564 (54.8)	219 (37.5)	
≥70 years	2479 (47.1)	2114 (45.2)	365 (62.5)	
ECOG PS				<.001
0-1	2714 (51.6)	2488 (53.2)	226 (38.7)	
2 or more	1358 (25.8)	1165 (24.9)	193 (33.0)	
Unknown	1190 (22.6)	1025 (21.9)	165 (28.3)	
Stage				<.001
I-III	1452 (27.6)	1324 (28.3)	128 (21.9)	
IV	3810 (72.4)	3354 (71.7)	456 (78.1)	
Treatment				<.001
No	720 (13.7)	566 (12.1)	154 (26.4)	
Yes (any)	4542 (86.3)	4112 (87.9)	430 (73.6)	

SCLC, small cell lung carcinoma; ECOG PS, Eastern Cooperative Oncology Group Performance Status.

[a]Comparison between smokers and never-smokers by Chi-square test.

© 2022 Tseng JS et al. *JAMA Network Open.*

eTable 2. Univariate analysis of characteristics between female smokers and never-smokers with small cell lung carcinoma from 2011 to 2018

Factors	All SCLC, No. (%) (n = 596)	Smokers, No. (%) (n = 322)	Never-smokers, No. (%) (n = 274)	*P* value[a]
Age				.27
<70 years	341 (57.2)	194 (60.2)	147 (53.6)	
≥70 years	255 (42.8)	128 (39.8)	127 (46.4)	
ECOG PS				.91
0-1	281 (47.1)	153 (47.5)	128 (46.7)	
2 or more	187 (31.4)	96 (29.8)	91 (33.2)	
Unknown	128 (21.5)	73 (22.7)	55 (20.1)	
Stage				.95
I-III	156 (26.2)	86 (26.7)	70 (25.5)	
IV	440 (73.8)	236 (73.3)	204 (74.5)	
Treatment				.97
No	109 (18.3)	60 (18.6)	49 (17.9)	
Yes (any)	487 (81.7)	262 (81.4)	225 (821)	

SCLC, small cell lung carcinoma; ECOG PS, Eastern Cooperative Oncology Group Performance Status.

[a]Comparison between smokers and never-smokers by Chi-square test.

© 2022 Tseng JS et al. *JAMA Network Open.*

eTable 3. Univariate analysis of characteristics between male and female never-smokers with small cell lung carcinoma from 2011 to 2018

Factors	Male, No. (%) (n = 584)	Female, No. (%) (n = 274)	*P* value[a]
Age			<.001
<70 years	219 (37.5)	147 (53.6)	
≥70 years	365 (62.5)	127 (46.4)	
ECOG PS			.10
0-1	226 (38.7)	128 (46.7)	
2 or more	193 (33.0)	91 (33.2)	
Unknown	165 (28.3)	55 (20.1)	
Stage			.50
I-III	128 (21.9)	70 (25.5)	
IV	456 (78.1)	204 (74.5)	
Treatment			.02
No	154 (26.4)	49 (17.9)	
Yes (any)	430 (73.6)	225 (821)	

SCLC, small cell lung carcinoma; ECOG PS, Eastern Cooperative Oncology Group Performance Status.

[a]Comparison between smokers and never-smokers by Chi-square test.

© 2022 Tseng JS et al. *JAMA Network Open.*

eFigure 1. Patient Selection and Analysis Flowchart

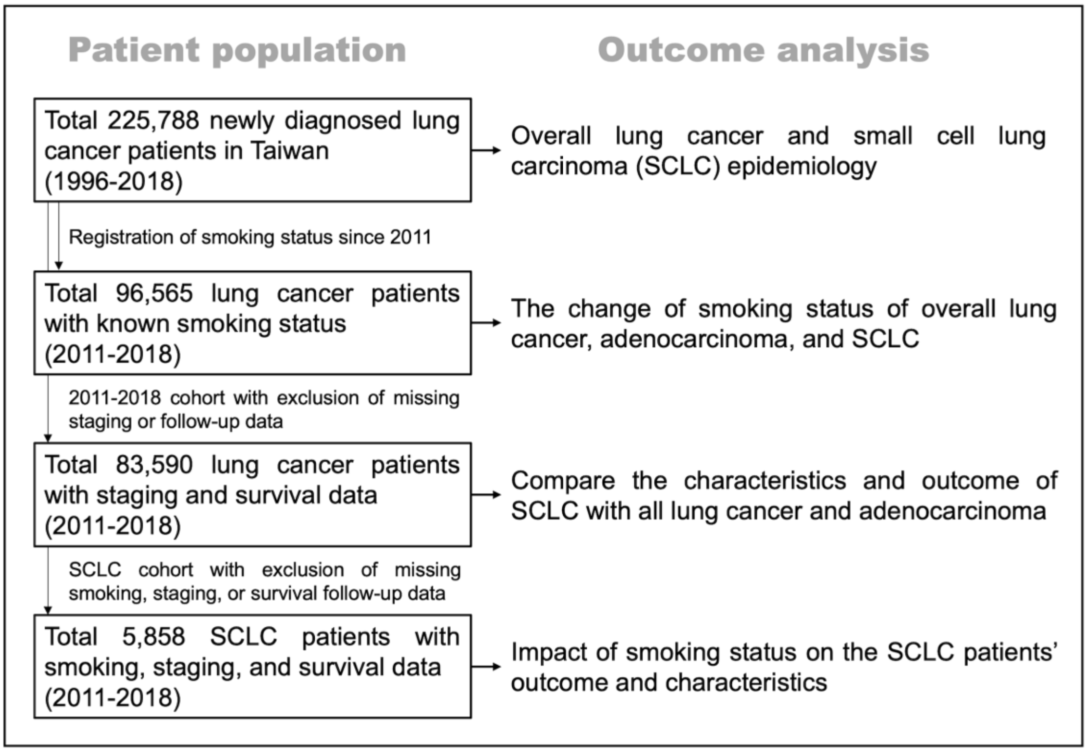

© 2022 Tseng JS et al. *JAMA Network Open.*

eFigure 2. Epidemiological Trends of Overall Lung Cancer and Small Cell Lung Carcinoma (SCLC) From 1996 to 2018 (n = 225 788)

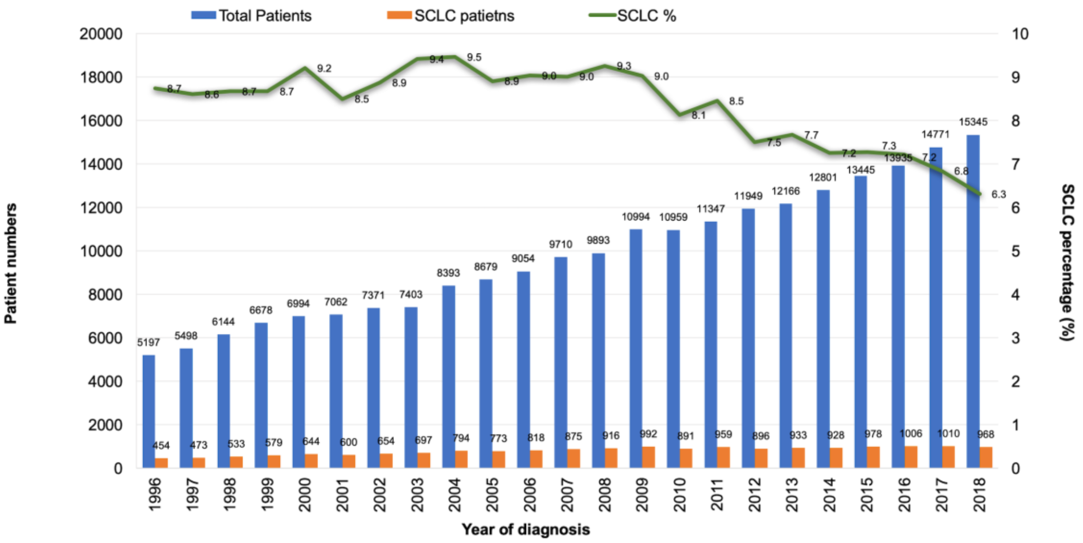

© 2022 Tseng JS et al. *JAMA Network Open.*

eFigure 3. Changes in Smoking Status Among All Lung Cancer Patients (A) and Patients With Lung Adenocarcinoma (B) From 2011 to 2018

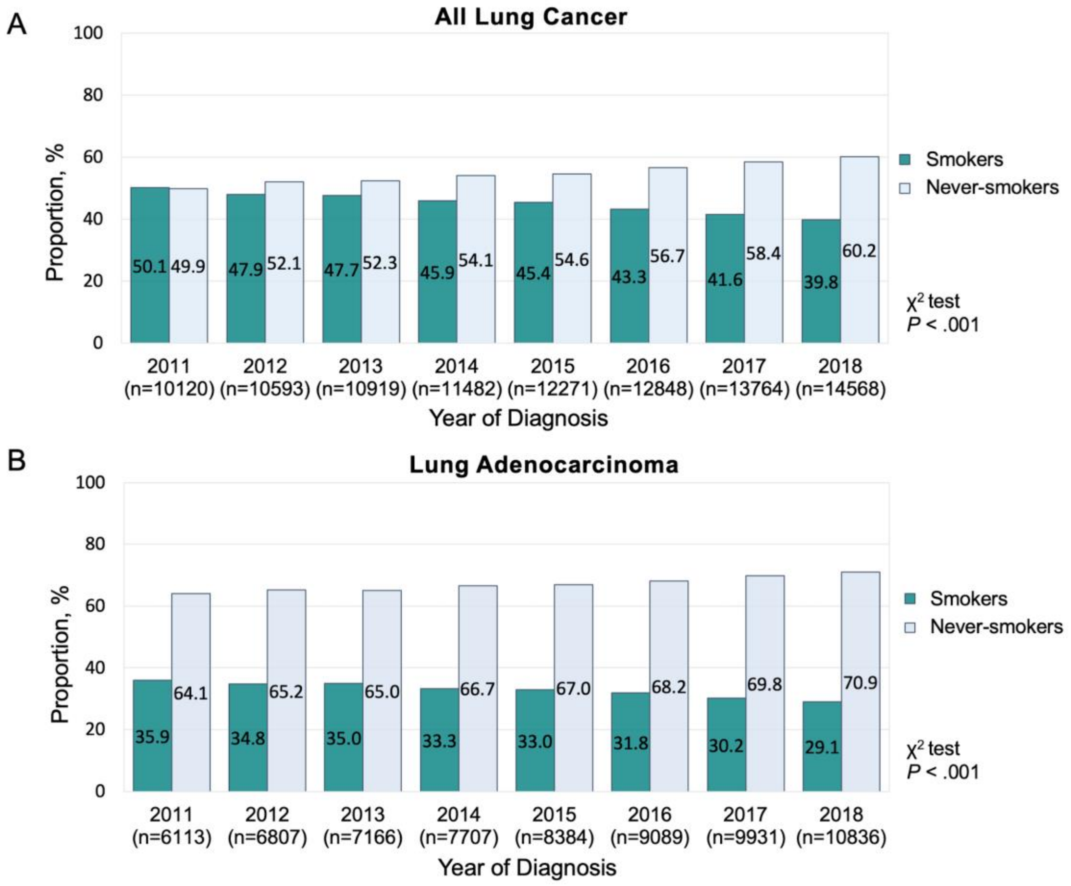

© 2022 Tseng JS et al. *JAMA Network Open.*

eFigure 4. Overall Survival of Patients With Small Cell Lung Carcinoma (SCLC) and Adenocarcinoma With Known Tumor Staging Data From 2011 to 2018

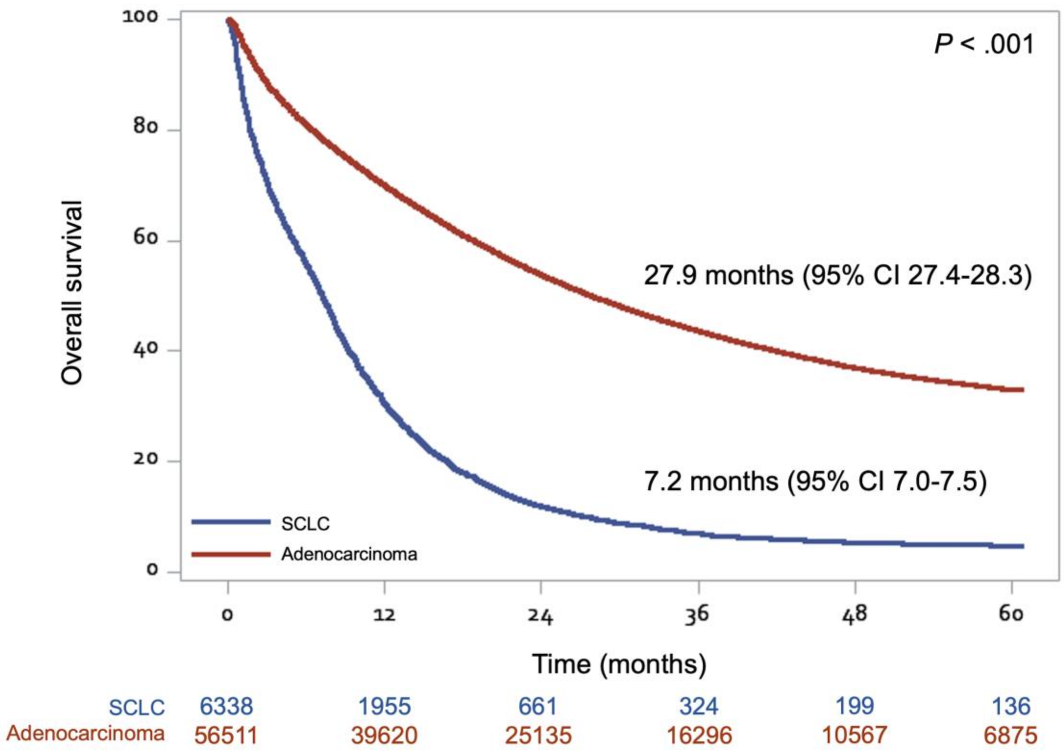

© 2022 Tseng JS et al. *JAMA Network Open.*

eFigure 5. Overall Survival of Patients With Small Cell Lung Carcinoma (SCLC) With Known Smoking Status and Tumor Staging Data From 2011-2018

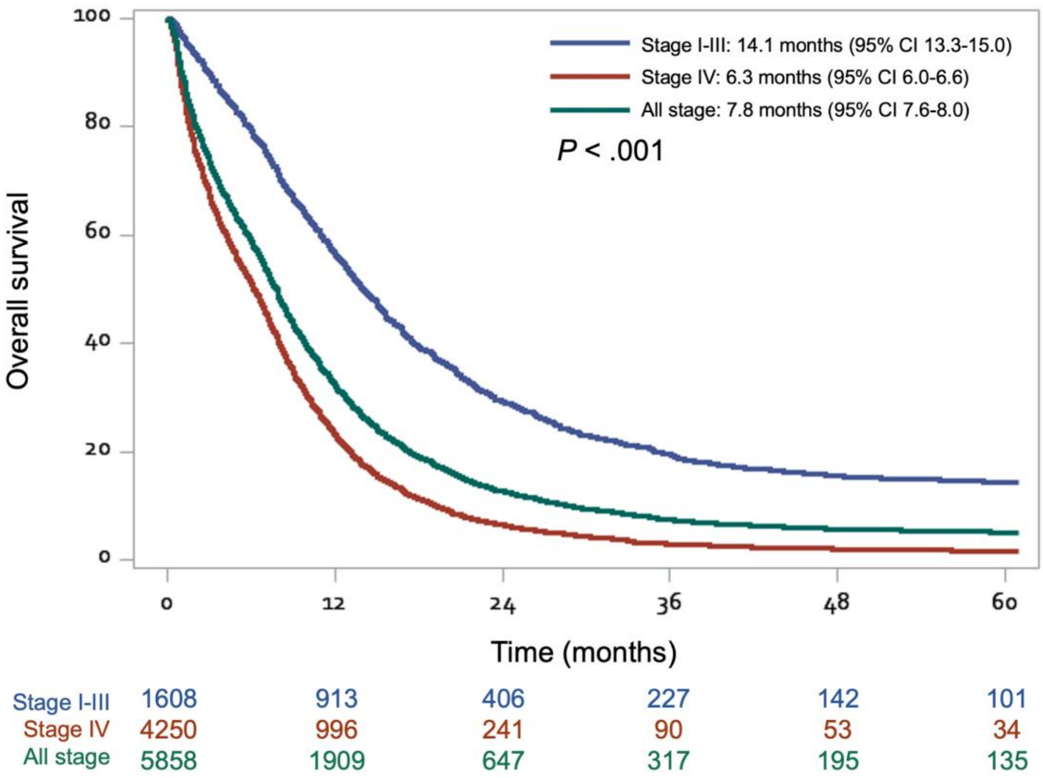

© 2022 Tseng JS et al. *JAMA Network Open.*

eFigure 6. Overall Survival of Smokers and Never-Smokers With Small Cell Lung Carcinoma (SCLC) in Stage I-III (A) and IV (B)

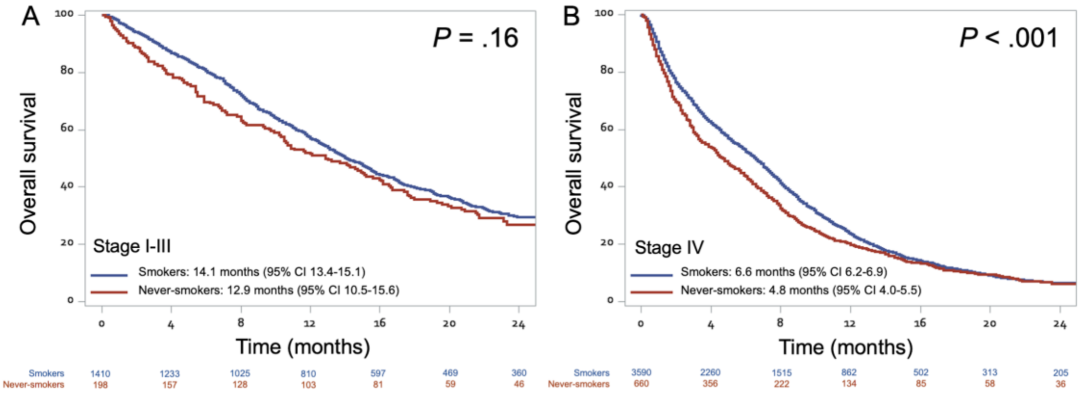

© 2022 Tseng JS et al. *JAMA Network Open.*
